# Awareness and Views of Pharmacists and Physicians Toward Prescribing of Drugs for Off-Label Use in the Pediatric Population in Saudi Arabia

**DOI:** 10.7759/cureus.23082

**Published:** 2022-03-11

**Authors:** Dawood Alyami, Ajaim A Alyami, Abdulaziz Alhossan, Mohammed Salah

**Affiliations:** 1 Department of Pharmacy, Riyadh Elm University, Riyadh, SAU; 2 Pharmaceutical Care Services, Khobash General Hospital, Najran, SAU; 3 College of Pharmacy, King Saud University, Riyadh, SAU

**Keywords:** off label prescription, pharmacist, physician, pediatric, children, off-label

## Abstract

Background: Prescribing off-label drugs in healthcare settings is a common practice worldwide. Thus, it is crucial to evaluate the knowledge and views of healthcare professionals regarding the off-label use of drugs in pediatric patients.

Methodology: A cross-sectional study was conducted from June to December, 2020, among physicians and pharmacists in the Kingdom of Saudi Arabia (KSA). A 28-item questionnaire was distributed to the healthcare professionals through an electronic link. Descriptive and inferential statistics were used to report the data.

Results: A total of 368 questionnaires were included in the final analysis. More than two-thirds of the respondents were aware of the correct definition of off-label drugs. Of the respondents, 37% were satisfied with their knowledge related to the use of off-label drugs in the pediatric population. Approximately half of the healthcare professionals in the study had major concerns about the efficacy and safety of off-label prescribing to children.

Conclusion: Off-label drug use among pediatric patients is common practice in the KSA and healthcare professionals providing treatment to the children are also aware of the concept of off-label drugs.

## Introduction

Prescribing drugs is recognized as a challenging practice that needs to be constantly monitored and refined. In addition, it is based on the knowledge of clinical pharmacology principles, knowledge about drugs, and particularly the experience of the prescribing physician [1,2]. Off-label prescribing is defined as “‘drugs prescribed and used for unapproved indications with respect to dosage, age, indication, or route” [3], whereas unlicensed drug use refers to ‘using drugs that are not specifically licensed for use in pediatric population’ [4]. Globally, prescribing off-label drugs in healthcare settings is a common practice [5]. The prescribing of off-label drugs is significantly more common than unlicensed drugs [6]. However, off-label prescribing is one of the challenges of prescribing in the pediatric population apart from the lack of randomized controlled trials in this age group and variation in drug disposing [5]. Technical constraints such as blood sampling are one of the significant factors that limit clinical studies in children [5].

When scientific and medical evidence justifies the use of the off-label drug, healthcare professionals promote the pediatric population’s interests by prescribing off-label drugs for their illnesses [7]. It is crucial to prescribe off-label drugs based on the degree of scientific evidence as it can also lead to patient harm [7]. A previous study of 160 commonly prescribed drugs concluded that a higher number of off-label drugs are prescribed with a lack of scientific support [8]. Likewise, patients are likely to receive harmful or ineffective treatment when drugs are irrationally prescribed or given without solid scientific evidence. Physicians typically follow empirical or experiential-based rationales when selecting the dose for children that attempts to achieve the same pharmacokinetic profile in children as in adults [9]. However, this approach may not be systematically explored during off-label use. Previous studies reported a higher incidence of adverse drug reactions and therapeutic failures due to the utilization of off-label or unlicensed drugs in the pediatric population [10]. Due to the difference in the body composition and development process in children, the scientific data of adults is appropriate for merely 6% of drugs prescribed to children [10].

Off-label prescriptions generally range from 10.5-80% and rates are relatively higher among the younger population and in the hospital settings [3,11-14]. A study in Britain revealed that one-third of all hospitalized pediatric patients received one or more off-label drugs [6]. Dosage was found to be the commonly reported off-label category in available literature [3,12,14]. In China, prescribing of off-label drugs was 46.9% and 53.9% in hospitalized populations and pediatric outpatients, respectively (Conference Paper: Guo Chun-ya. A Survey of Off-label Drug Use Prescriptions in Outpatient of Beijing Children's Hospital; 2014), [15]. Globally, there is a lack of harmonization related to the practice of prescribing off-label drugs [16]. Developed countries such as France, Britain, and United States have established national legislations and guidelines for off-label drugs for promoting rational prescribing of these drugs [17-19]. whereas the practice of off-label prescribing is illegal in India [20]. In the Kingdom of Saudi Arabia (KSA), it is mandatory to solicit approval from the Saudi Food and Drug Administration for all drugs that ensures the efficacy, safety, and quality of marketed drugs [21]. However, there is no clear description according to the Saudi Ministry of Health and Saudi Food and Drug Administration. Healthcare settings in the KSA are obligated to follow the standards of the Saudi Central Board for Accreditation of Healthcare Institutions (CBAHI). CBAHI standards for medication management (MM 24) states that “the hospital has a system for prescribing non-formulary medications and prescribing formulary medications for off-label (unapproved) indication or investigation” [22].

There are several strategies such as protocols, legislation, procedures, and local guidelines, that are implemented for intended benefits and reducing the negative impact of off-label prescribing, while knowledge and prescribing practices are recognized as crucial factors for the successful implementation of these strategies. However, there are no guidelines regarding off-label prescribing in the KSA. Since off-label drugs are associated with both benefits and harm, it is all the more important to evaluate the awareness and current practices of healthcare professionals towards off-label prescribing in the pediatric population. Little is known about the awareness and views of physicians and pharmacists towards off-label prescribing in the KSA. Therefore, we aimed to assess the knowledge and views of pharmacists and physicians regarding off-label drugs in the pediatric population. In addition, we also explored the dosing issues related to off-label drugs. The findings of this study will help alleviate this scarcity in literature and aid in the implementation of awareness and educational programs, thus improving the prescribing of off-label prescribing and the safety of the pediatric population.

## Materials and methods

A cross-sectional survey was conducted from June to December, 2020, for assessing the awareness and views of licensed physicians and pharmacists (working in the central, eastern, western, and northern regions of the KSA) regarding off-label drugs in the pediatric population. To elicit a response in 54% of respondents regarding awareness of off-label drugs, with a 95% CI, a sample size of 375 was estimated.

A 28-item questionnaire was designed to explore awareness and views regarding off-label drugs. The self-administered questionnaire was adapted from previous studies [16]. The questionnaire comprises four sections such as general information of participants, awareness of healthcare professionals about off-label drugs, experiences, and views of healthcare professionals about off-label drugs, and dosing issues related to off-label drugs. A web link to the survey questionnaire was distributed to licensed physicians and pharmacists working in private and government hospitals throughout the KSA. The study objectives were included on the first page of the questionnaire under the consent statement to provide general information to the study participants.

A waiver of written informed consent was granted by the ethics committee and this non-invasive study was initiated after obtaining approval from the Institutional Review Board of the Riyadh Elm University (approval number: FPGRP/2020/488/322/312).

The sample size was calculated by an online sample size calculator [23]. Data analysis was carried out using the IBM SPSS Statistics for Windows, Version 22.0 (Released 2013. IBM Corp., Armonk, New York, United States). Descriptive statistics were used to report the data. Age was presented in mean ± SD, median, and range whereas categorical study variables such as gender, level, and category of the hospital, city of the hospital, specialty, years of experience, responses to questions regarding awareness, experiences, and dosing-related issues on off-label use were described in frequencies and percentages. 

## Results

A total of 368 participants submitted questionnaires through the website of SurveyMonkey® ((Momentive Inc., San Mateo, California, United States). Many of the respondents (n=242, 65.8%) were male (Table 1). Likewise, the majority (85.1%) of the respondents were less than 40 years old. However, the mean age of the study participants was 32.6 (+7.2). Of the participants, 41% belonged to the tertiary care setting. Similarly, 103 (28%) and 113 participants (30.7%) were affiliated with the primary care and secondary care settings, respectively (Table 1). Participants responded from different cities of the KSA such as Riyadh, Jeddah, Tabuk, Dammam, Makkah, Madinah, and Taif; some also responded from the smaller cities. Participants of different specialties responded to our study questionnaires such as general practitioners (5.2%), pediatricians (9.5%), specialists (4.6%), pharmacists (61.1%), and clinical pharmacists (9.8%). The majority of the healthcare professionals had one to four years of working experience (n=146, 39.7%).

**Table 1:** Study demographics (n=368) SD=standard deviation. All numerical data presented in mean ±SD. All categorical data are presented in n (%).

Variables	Overall (n=368)
Age, years	
Mean ±SD	32.6 ±7.2
Min – Max	20 – 62
Median	32
Gender	
Male	242 (65.8)
Female	126 (34.2)
Age groups	
< 40 years	313 (85.1)
≥ 40 years	55 (14.9)
Level of Hospital	
Primary	103 (28)
Secondary	113 (30.7)
Tertiary	152 (41.3)
City of Hospital	
Riyadh	81 (22)
Jeddah	46 (12.5)
Tabuk	7 (1.9)
Dammam	19 (5.2)
Makkah	28 (7.6)
Madinah	15 (4.1)
Taif	16 (4.3)
Others	156 (42.4)
Specialty	
General Practitioner	19 (5.2)
Pediatrician	35 (9.5)
Specialist	17 (4.6)
Pharmacist	225 (61.1)
Clinical Pharmacist	36 (9.8)
Others (nurses)	36 (9.8)
Years of Experience	
1-4 years	146 (39.7)
5-9 years	89 (24.2)
10-14 years	79 (21.5)
≥15 years	54 (14.7)

**Figure 1:**
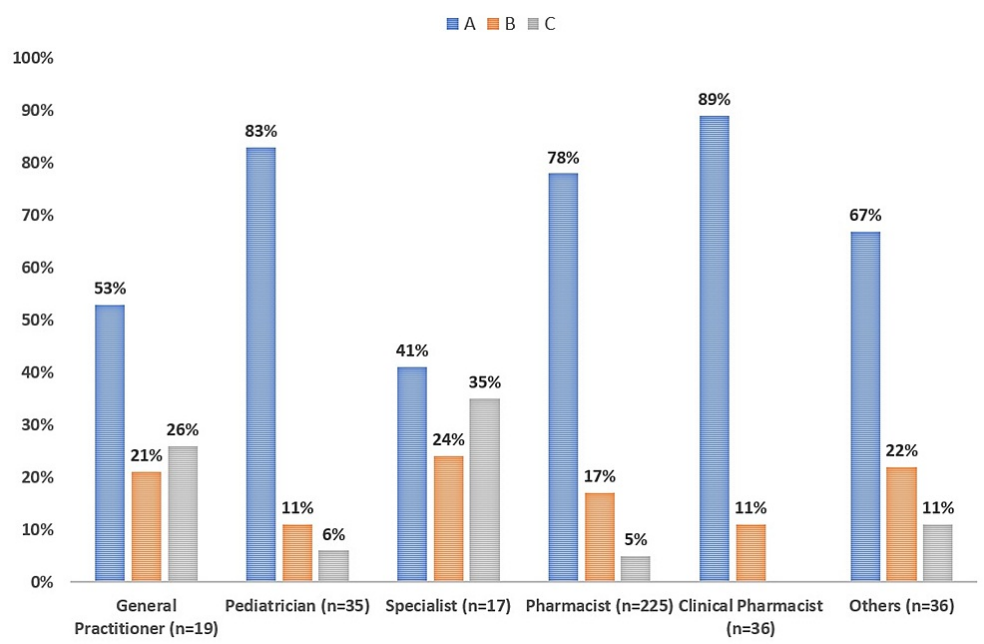
Percentage of off-label drug definitions as per specialty A=Drugs prescribed and used outside their licensed indications with respect to dosage, age, indication or route. B=Drugs that are not approved for any indication by the Saudi Food and Drug Administration. C=None of the above.

**Figure 2:**
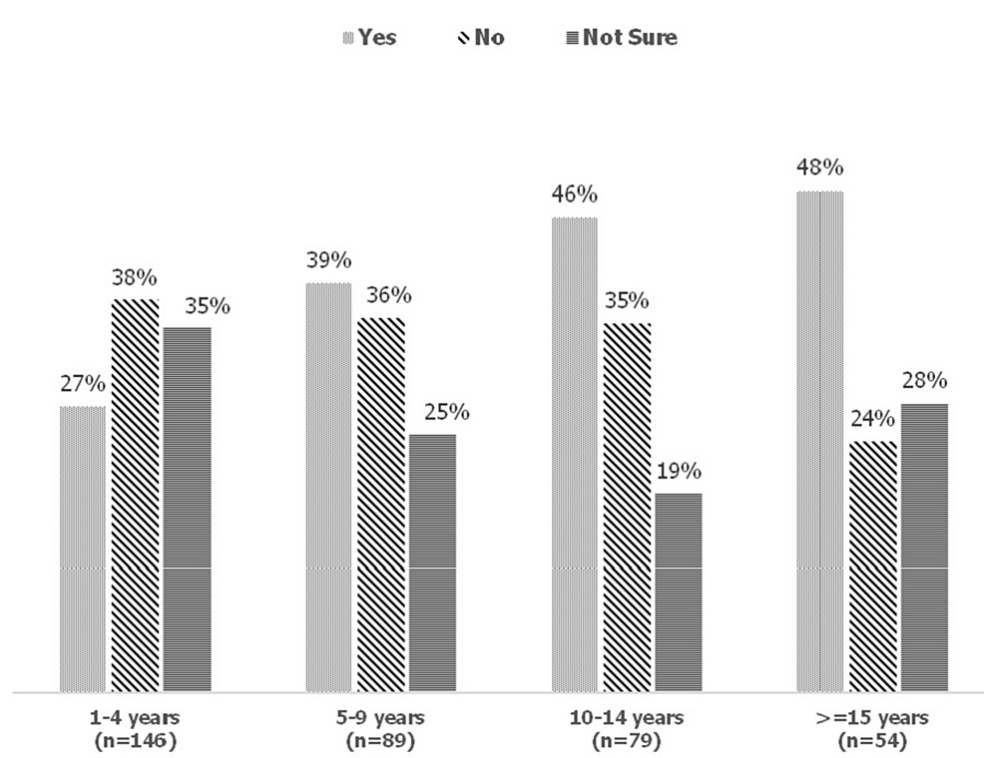
Percentage of adequate knowledge about the use of off-label drugs in pediatrics as per healthcare professionals’ years of experience.

Figure 1 depicts the percentage of off-label drug definitions as per specialty. Figure 2 illustrates the percentage of adequate knowledge about the use of off-label drugs in pediatrics as per years of experience.

Table 2 demonstrates the awareness of physicians and pharmacists regarding off-label prescribing. More than two-thirds of the respondents were aware of the correct definition of off-label drugs. In contrast, 25% of respondents were not aware of the correct definition of off-label drugs. Of the respondents, 37% were satisfied with their knowledge related to the use of off-label drugs in the pediatric population. However, one-third of the participants were not satisfied with their knowledge related to the use of off-label drugs in children. According to the healthcare professionals, adverse reactions (77%) are the most common risk associated with the use of off-label drugs, followed by inefficiency (47.6%), improper formulations (30.4%), an increase of therapeutic errors (59.2%), and no risk (6.3%).

**Table 2:** Awareness of physicians and pharmacists regarding off-label prescribing (n=368) All categorical data are presented in n (%).

Variables	Overall
Off-label drug is defined as	
Drugs prescribed and used outside their licensed indications with respect to dosage, age, indication or route.	276 (75)
Drugs that are not approved for any indication by the Saudi Food and Drug Administration.	63 (17.1)
None of the above.	29 (7.9)
Do you consider your knowledge is adequate about the use of off-label drugs in pediatrics?	
Yes	137 (37.2)
No	128 (34.8)
Not sure	103 (28)
What are the risks associated with the use of off-label drugs?	
Adverse reactions	286 (77.7)
Inefficiency	175 (47.6)
Improper formulations	112 (30.4)
Increase of therapeutic errors	218 (59.2)
No risk	23 (6.3)
What is the process of off-label drug use in your hospital?	
Applying with relative information and evidence	142 (38.6)
Being approved by the ethics committee	76 (20.7)
Being approved by the pharmacy and therapeutic committee	198 (53.8)
Obtaining informed consent	62 (16.8)
Monitoring the adverse reaction	102 (27.7)
Do not know	85 (23.1)
Is there any law or regulation regarding off-label drugs in the Kingdom of Saudi Arabia?	
Yes	171 (46.5)
No	39 (10.6)
Don’t know	158 (42.9)
Do you know about the standards related to off-label drugs provided by Saudi Central Board for Accreditation of Healthcare Institutions (CBAHI)?	
Yes	148 (40.2)
No	96 (26.1)
Don’t know	124 (33.7)

The majority (n=198, 53.8%) of the respondents were of the view that off-label drugs are approved by the pharmacy and therapeutic committee, while 23% were not aware of the process of off-label drug use in their hospital. A total of 171 (46.5%) professionals believed that there is a law or regulation regarding off-label drugs in the KSA. Approximately 53% of the healthcare professionals were not aware of any law or regulation regarding off-label drugs in the KSA. Many participants (40.2%) were aware of the standards related to off-label drugs provided by the Saudi CBAHI.

Table 3 demonstrates the experiences and views of physicians and pharmacists regarding off-label medicines for children. Of the participants, 43% heard about off-label drugs for the first time during their undergraduate studies while 31% heard about off-label drugs during their employment. Interestingly, 9.5% of participants did not hear about off-label drugs before filling out our survey questionnaire. Approximately half of the healthcare professionals had major concerns about the efficacy of off-label prescribing to children and 12% did not show any concern about it. Similarly, 54.6% of the healthcare professionals had major concerns about the safety of off-label prescribing to children and 32 (8.7%) professionals did not show any concern about this.

**Table 3:** Experiences and views of physicians and pharmacists regarding off-label medicines for children (n=368) All categorical data are presented in n (%).

Variables	Overall
When did you first time hear about off-label drugs?	
During undergraduate studies	159 (43.2)
During post-graduate studies	60 (16.3)
Through my experience	114 (31)
Terminology is new to me	35 (9.5)
Frequency of off-label prescription to children during the previous year	
≤10%	182 (49.5)
11% to 25%	142 (38.6)
≥ 26%	44 (12)
Are there any concerns about the efficacy of off-label prescribing to children?	
Yes, major concerns	182 (49.5)
Yes, minor concerns	142 (38.6)
No, no concerns at all	44 (12)
Are there any concerns about the safety of off-label prescribing to children?	
Yes, major concerns	201 (54.6)
Yes, minor concerns	135 (36.7)
No, no concerns at all	32 (8.7)
Did you ever experience an adverse drug reaction due to off-label drugs among children?	
Yes	104 (28.3)
No	264 (71.7)
Did you ever experience treatment failure following off-label prescribing of medicines to children?	
Yes	127 (34.5)
No	241 (65.5)
Most likely recipient of off-label prescriptions among children	
Neonates, including preterm and term from birth to 28 days	62 (16.8)
Infants (1-23 months old)	80 (21.7)
Children 2-11 years old	136 (37)
Children 12-16 years old	90 (24.5)
Should parents be informed about off-label prescription to children?	
Yes	292 (79.3)
No	76 (20.7)
What is the most common category of off-label prescribing in children?	
Age	92 (25)
Indication	161 (43.8)
Dosage	80 (21.7)
Route of administration	33 (9)
Other	2 (0.5)
Is there any guideline or policy of off label use in your hospital?	
Yes	177 (48.1)
No	88 (23.9)
Don’t know	103 (28)
Is it necessary for pharmacists to intervene when off-label drug use happen?	
Yes	294 (79.9)
No, it intervenes the normal routine	74 (20.1)

Nearly 72% of respondents did not experience any adverse drug reaction due to off-label drug prescription to children (Table 3). Two-thirds of the respondents experienced treatment failure following off-label prescribing of medicines to children. Of the participants, 37% were of the view that children in the age group of 2-11 years were the more likely recipient of off-label drugs prescriptions, followed by those in the age group of 12-16 years (24.5%), infants aged in the age group of 1-23 months (21.7%), and neonates, including preterm and term from birth to 28 days (16.8%) (Table 3). Approximately 80% of professionals believed that parents should be informed about off-label prescriptions to children. According to the responses, indication (43.8%) was found to be the most common category of off-label prescribing in children, followed by age (25%), dosage (21.7%), and route of administration (9%).

Nearly half of the healthcare professionals were of the view that there is a guideline or policy of off-label use in their hospital. Interestingly, 79.9% of the respondents believed that it is necessary for pharmacists to intervene when off-label drug use happens.

Table 4 highlights dosing issues related to off-label drugs. The majority of the participants reported that they regularly double-check doses of medicines used in children if a calculation was required in deciding on the dose to be given. A total of 312 (84.8%) participants reported the occurrence of dosing errors in the pediatric population. More than two-thirds of the participants were of the view that licensing more medicines for use in children will decrease dosing errors in pediatrics.

**Table 4:** Dosing issues related to off-label drugs (n=368) All categorical data are presented in n (%).

Variables	Overall
Do you routinely double-check doses of medicines used in children if a calculation was required in deciding on the doses to be given?	
Yes	311 (84.5)
No	34 (9.2)
Don’t know	23 (6.3)
Dosing error occurred in a pediatric prescription	
Yes	312 (84.8)
No	56 (15.2)
Do you think that licensing more medicines for use in children will decrease dosing errors in pediatrics (Testing medicines for their off-label indications and adding those indications to the label)?	
Yes	254 (69)
No	53 (14.4)
Do not know	61 (16.6)

## Discussion

In the KSA, and globally, healthcare professionals are increasingly put under ethical and professional obligations for ensuring safe medication therapy. Thus, it is crucial to ascertain the issues surrounding off-label prescribing. As per our knowledge, this is the first study assessing the awareness and views of healthcare professionals in the KSA regarding off-label drugs in pediatric patients. We contemporaneously explored a range of healthcare providers in the KSA in terms of their views pertinent to the prescribing of off-label drugs in the pediatric population and the current management situation.

We found that some of the results were concordant with the findings of previous studies carried out in other countries, such as the use of off-label drugs was prevalent and that a large number of healthcare providers were familiar with the concept of the use of off-label drugs [16,24]. A large number of the study participants realized that there are major safety concerns of off-label prescribing to children, which are in line with earlier findings [16]. Off-label drugs not only carries risk, but it also possesses several benefits, especially when patients have exhausted all other available approved therapies [25]. The majority of the respondents reported that it is essential to inform parents of children about the prescribing of off-label drugs. This finding is also consistent with earlier reports [16].

In several other countries, the practice of prescribing off-label drugs is legal [17-19]. However, there is no such official law or regulation in the KSA related to the prescribing of off-label drugs have been identified that may potentially create complex ethical and legal situations especially pertinent to medical liability. Surprisingly, half of the healthcare professionals in this study were of the view that there is a law in the KSA in this regard. After indication, a common reason for off-label prescribing in pediatric patients was dosage, which contrasts with the results of a recently published Chinese study [16].

The most important strength of this study was the diversity among healthcare professionals who responded to the study questionnaire from different regions of the KSA. However, there are certain limitations as well. The nature of this study meant that we superficially explored this research area and more detailed work would be of value in the future, including exploring off-label practice nationally and strategies to overcome barriers. Another limitation is related to the self-reported questionnaire as outcomes may be certainly influenced by healthcare professionals’ subjective factors. However, it is anticipated that the anonymity of the study questionnaire urged honesty. The recommendations are presented in Table 5.

**Table 5:** Recommendations for improving the prescribing of off-label drugs and safety of the pediatric population KSA: Kingdom of Saudi Arabia

Recommendations	
1	It is recommended to conduct more studies and clinical trials to support the marketing of specific drugs for children.
2	It is crucial to improve the training of doctors on the subject and make them well prepared in prescribing off-label drugs to the pediatric population, thus reducing safety-related issues.
3	It is also recommended to review the curriculum and training programs of medical and pharmacy students to include important issues including the treatment of pediatric patients.
4	Since there are no official guidelines in the KSA for the off-label use of drugs in children, there is a need to develop guidelines for improving medication safety in a pediatric population.
5	There is also a need to develop a post-marketing drug surveillance system for measuring the drug effectiveness and risks in routine clinical practice, thus securing off-label drug prescriptions that involve major adverse effect risks.

## Conclusions

Off-label drug use among pediatric patients is common practice in the KSA and healthcare professionals providing treatment to the children are also aware of the concept of off-label drugs. Many healthcare professionals had major concerns related to the efficacy and safety of off-label drugs. As a baseline study in the KSA, our findings should be of interest to the off-label stakeholders, particularly physicians, for conducting customized training programs and introducing official regulations related to the practice of prescribing off-label drugs in the pediatric population, thus promoting safety and improving pediatric patients’ outcomes.
